# Predicting Factors of Clinical Outcomes in Traumatized Adults and Older Adults

**DOI:** 10.1093/geroni/igab046.2285

**Published:** 2021-12-17

**Authors:** Pornthip Suyasith, Prangtip Chayaput, Orapan Thosingha, Suzanne G Leveille, Jatuporn Sirikun

**Affiliations:** 1 University of Massuchesetts Boston, Bangkok, Samut Sakhon, Thailand; 2 Faculty of Nursing, Mahidol University, Bangkoknoi, Bangkok, Samut Sakhon, Thailand; 3 Faculty of Nursing, Chulabhorn Royal Academy, Laksi, Bangkok, Samut Sakhon, Thailand; 4 CNHS, UMass Boston, CNHS, UMass Boston, Massachusetts, United States; 5 Faculty of Medicine Siriraj Hospital, Bangkoknoi, Bangkok, Samut Sakhon, Thailand

## Abstract

To investigate factors predicting hospital mortality and hospital length of stay (LOS) in traumatized adults and older adults, we conducted a three-year retrospective study at an academic hospital, Bangkok, Thailand. We reviewed medical records of 627 trauma patients admitted to the ED. Subjects were classified into 2 groups: adults (□55y), and older adults (□55y). Data were collected for demographic and clinical characteristics, physiologic deterioration using the Modified Early Warning Score (MEWS), severity of injury using the Circulation Respiration Abdomen Motor and Speech Score (CRAMS), and outcomes of hospital mortality and LOS. Multivariable logistic and linear regression models were performed. For hospital mortality, an elevated MEWS (Older adults [n= 267]: MEWS≥3, OR=4.80, 95%CI, 1.02-22.56 vs Adults [n = 360]: MEWS≥4, OR=11.63, 95%CI, 1.94-69.82) and CRAMS (Older adults: CRAMS≤9, OR=19.21, 95%CI, 2.78-132.98 vs Adults: CRAMS≤6, OR=18.58, 95%CI, 3.40-101.65) were strongly predictive, adjusted for demographic and clinical data. For LOS, road traffic accident (RTA) (Older adults: β=0.80, 95%CI, 0.31-1.29, p < .01 vs Adults: β=0.44, 95%CI, 0.21-0.67, p < .001) and falls (Older adults: β=0.88, 95%CI, 0.44-1.32, p < .001 vs Adults: β=0.33, 95%CI, 0.02-0.65, p < .05) were associated with LOS, adjusted for demographic and clinical data. MEWS and CRAMS predicted hospital mortality, and RTA and falls predicted LOS in both age groups. Results support the need for interventions for close monitoring and medical management for older traumatized patients based on CRAMS and MEWS assessment to decrease the risk of death, and targeting those sustaining falls and RTA to reduce prolonged LOS.

